# Advanced Diagnostic Methods in Necrotizing Sialometaplasia of the Parotid Glands: An Updated Literature Review and a Rare Case Report

**DOI:** 10.3390/jcm14072290

**Published:** 2025-03-27

**Authors:** Rares Mocan, Cristian Dinu, Sebastian Stoia, Grigore Baciut, Simion Bran, Manuela Lenghel, Tiberiu Tamas, Gabriel Armencea, Emil Botan, Florin Onisor, Mihaela Baciut

**Affiliations:** 1 Department of Maxillofacial Surgery and Implantology, Faculty of Dentistry, “Iuliu Hațieganu” University of Medicine and Pharmacy, 400012 Cluj-Napoca, Romania; mocanrares@gmail.com (R.M.); stoia_sebi@yahoo.com (S.S.); gbaciut@umfcluj.ro (G.B.); dr_brans@yahoo.com (S.B.); tibi.tamas@yahoo.com (T.T.); garmencea@gmail.com (G.A.); florin.onisor@gmail.com (F.O.); mbaciut@yahoo.com (M.B.); 2 Department of Radiology, Faculty of Medicine, “Iuliu Hațieganu” University of Medicine and Pharmacy, 400012 Cluj-Napoca, Romania; lenghel.manuela@gmail.com; 3 Department of Pathology, Emergency County Hospital, 600114 Cluj-Napoca, Romania; emilbotan@gmail.com

**Keywords:** necrotizing sialometaplasia, parotid gland, multiparametric magnetic resonance imaging

## Abstract

**Background/Objectives**: Necrotizing sialometaplasia (NS) is an inflammatory condition of the salivary glands that can closely mimic malignancy. While it predominantly affects the minor salivary glands of the hard palate, it can also occur in the parotid gland, leading to potential misdiagnosis and unnecessary treatment. This study aims to analyze the characteristics, diagnostic challenges, and management of parotid gland NS through a comprehensive literature review and a case report. **Methods**: A systematic literature review was conducted using PubMed, including all relevant publications up to December 2024. The research strategy focused on cases of NS affecting the parotid gland. Additionally, a rare case of misdiagnosed parotid NS is presented to highlight clinical and diagnostic challenges. **Results**: The review identified 30 patients, with a mean age of 49 years, and a nearly equal distribution between sexes. Their etiology was primarily linked to vascular insufficiency, often triggered by surgical trauma (20 cases), tumors (1 case), vascular fragility (4 cases), smoking (1 case), or other factors (4 cases). Clinically, NS can resemble squamous cell carcinoma, presenting with neck swelling, pain, and imaging features suggestive of malignancy. Histopathological assessment remains the gold standard for diagnosis. A rare case of a 23-year-old female misdiagnosed with squamous cell carcinoma emphasizes the importance of multiparametric MRI imaging and histological re-evaluation in atypical presentations. **Conclusions**: NS of the parotid gland is a rare but significant diagnostic challenge, due to its resemblance to malignant tumors. While conventional imaging may suggest a neoplastic process, advanced techniques such as DWI (Diffusion-Weighted Imaging) and ADC (Apparent Diffusion Coefficient) mapping can offer valuable noninvasive insights. A multidisciplinary approach, incorporating clinical history, imaging, and histopathological assessment, is essential in order to avoid unnecessary treatment. In cases where NS is suspected, a conservative approach with careful follow-up may be warranted to prevent overtreatment.

## 1. Introduction

Necrotizing sialometaplasia is an inflammatory disease of the salivary glands that is capable of mimicking malignancy. It is usually found in the palate, but can be diagnosed in any salivary gland, including the parotid gland [1].

On the basis of a histopathological misdiagnosis, unnecessary or inappropriate therapy can be conducted by clinicians [2].

An ischemic basis, such as a traumatic injury or medical procedures, is the most accepted cause for this disease, although its exact etiology has not been fully elucidated [3]. Within the parotid gland, the basic histopathologic lesion is represented by the infarction of the salivary lobules, with subsequent repair and metaplasia [4].

It primarily affects the hard palate’s minor salivary glands, with rare occurrences in the parotid gland (estimated incidence of approximately 10%) [5].

Multiparametric MRI has never been explored as a diagnostic tool for necrotizing sialometaplasia, despite its ability to distinguish benign from malignant lesions. This study evaluates the potential of Diffusion-Weighted Imaging and Apparent Diffusion Coefficient mapping in identifying ischemic changes characteristic of NS. Given the frequent misdiagnosis of NS as salivary gland carcinoma, multiparametric MRI may improve diagnostic accuracy and prevent unnecessary surgical interventions.

## 2. Materials and Methods

**Figure 1:**
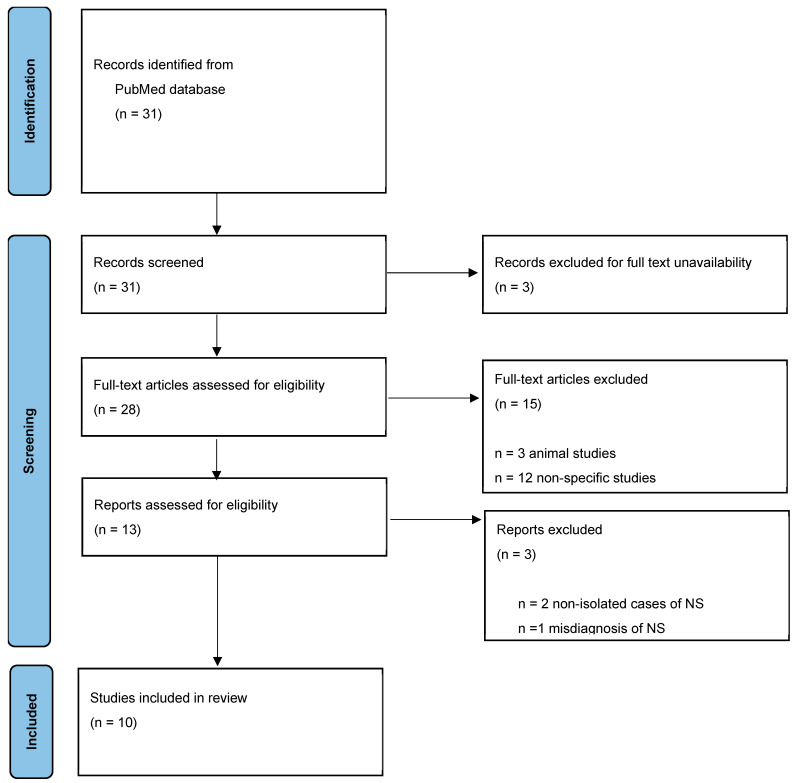
Study selection process.

A comprehensive literature search was conducted using PubMed, covering all publications up to December 2024. The search strategy included the keywords ‘necrotizing sialometaplasia’ and ‘parotid gland’. The Pubmed query was as follows: (“parotid gland”[MeSH Terms] OR (“parotid”[All Fields] AND “gland”[All Fields]) OR “parotid gland”[All Fields]) AND (“necrotising sialometaplasia”[All Fields] OR “sialometaplasia, necrotizing”[MeSH Terms] OR (“sialometaplasia”[All Fields] AND “necrotizing”[All Fields]) OR “necrotizing sialometaplasia”[All Fields] OR (“necrotizing”[All Fields] AND “sialometaplasia”[All Fields])). The eligibility criteria were restricted to articles reporting cases of necrotizing sialometaplasia affecting the parotid gland. Studies that were animal-based or otherwise ineligible were excluded (Figure 1). The characteristics of the included papers were determined by two reviewers (L.M. and E.B.).

The data extracted included patient demographics, lesion characteristics, diagnostic pathways, and treatment outcomes. The findings were synthesized narratively, given the heterogeneity of the included studies.

## 3. Results

### 3.1. Epidemiology

The literature review showed the mean age of male participants diagnosed with necrotizing sialometaplasia was 52.17 years, and for females, it was 45.92 years, with an overall mean age of 49 years.

The age range spanned from 17 to 83 years, demonstrating a relatively equal distribution across the ages, with no significant skew bias toward any particular sex (Table 1).

### 3.2. Etiology

Manning et al. reported a strong association between parotid gland NS and prior surgery for a different parotid tumor. In seven out of eight cases, NS symptoms developed within 3.5 weeks of the initial procedure [1]. Some authors affirm that the most common cause of necrotizing sialometaplasia of the salivary glands is trauma caused by dental procedures [5], while most affirm that the etiology is thought to be an insufficient blood supply arising from a large variety of other causes [4]. Other suspected causes are a tumor [8], vascular fragility [3], pressure-induced ischemia [7], vascular injury [2], heavy smoking [9], and a spontaneous hematoma due to an overdose of anticoagulant treatment [11].

**Figure 2:**
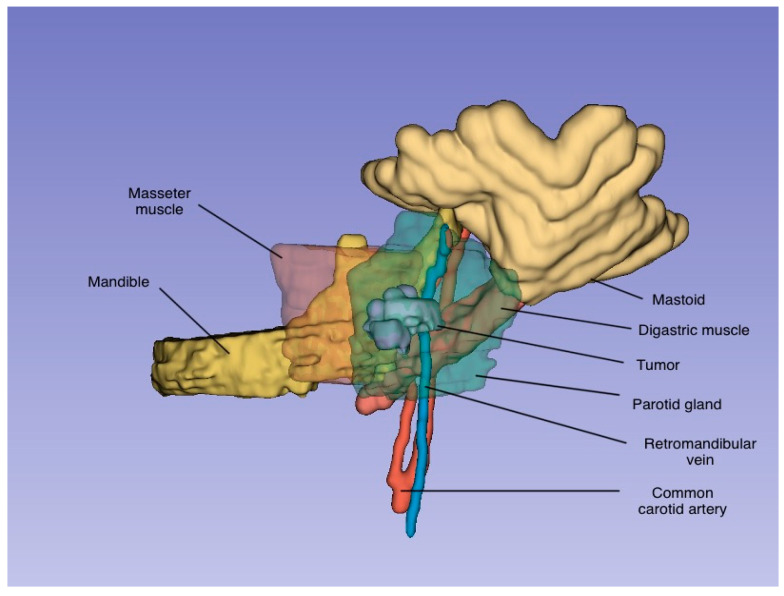
A three-dimensional representation of the tumor and the surrounding anatomical structures, generated using 3D Slicer, an open-source platform for medical image processing and visualization.

A disruption in the blood supply to a salivary gland can lead to necrotizing sialometaplasia, as the blood vessels supplying the glands are particularly susceptible due to their passage through foramina, making them vulnerable to injury. In major salivary glands, the arteries accompany the subdivisions of the secretory duct system, ensuring that each salivary lobule has a separate and distinct blood supply [13]. Standish and Shafer [14] demonstrated the development of necrotizing sialometaplasia in the salivary glands of rats following the experimental ligation of these vessels. The characteristic lobular pattern observed in necrotizing sialometaplasia is attributed to the distinct blood supply to each lobule (the distribution pattern of the tumor is presented in Figure 2).

Some authors have described the presence of NS in association with different salivary gland tumors: epithelial–myoepithelial carcinomas, adenoid cystic carcinomas [15], Warthin tumors, and oncocytomas [16].

### 3.3. Clinical Features

Several cases are reported in the literature that affirm the close resemblance of necrotizing sialometaplasia to squamous cell carcinoma and mucoepidermoid carcinoma [5].

The clinical and histological presentation of NS can vary, likely reflecting the different stages of the disease process, from initial infarction to healing [17]. NS often resolves spontaneously within 3 to 12 weeks without treatment [5].

Neck swelling is the most frequently reported symptom, followed by pain, pus discharge from the salivary duct, and in one case, pharyngeal swelling, vocal cord paralysis, and neck lymphadenopathy. The reported sizes are between 6 × 10 mm and 40 × 50 mm (Table 1).

### 3.4. Diagnosis

To date, necrotizing sialometaplasia has not been definitively identified using biopsy techniques. Fine-needle aspiration (FNA) typically reveals epithelial and inflammatory cells; however, even when squamous cells lack atypia, it remains challenging to confidently conclude an “inflammatory lesion”. Similarly, Tru-Cut biopsies depend heavily on the puncture trajectory, often failing to clarify the extent to which the architecture is preserved. Open incisional biopsy presents similar limitations, as it may not provide a full representation of the lesion’s structural context.

Necrotizing sialometaplasia can be difficult to diagnose definitively, as both squamous metaplasia and necrosis are also seen in various inflammatory and neoplastic salivary gland conditions [18]. Specific microscopic criteria are essential for the accurate diagnosis of NS, including lobular necrosis of salivary tissue, variable presence of granulation tissue, acute and chronic inflammation, squamous metaplasia within ductal and/or acinar structures, and preservation of the overall salivary gland lobular architecture.

### 3.5. Radiology Assessment

While initial imaging studies (ultrasound, CT, MRI) often raise suspicion of malignancy due to the tumor-like appearance of NS lesions, these modalities also help to localize the lesion, delineate its extent, and evaluate surrounding structures. Specific imaging features, such as areas of necrosis, surrounding inflammation, and a potential lack of aggressive characteristics, can offer clues pointing towards NS. While gallium scintigraphy might suggest a non-malignant etiology [19], the definitive diagnosis of NS relies heavily on histopathological assessment. Nevertheless, careful interpretation of imaging findings is a valuable tool to guide diagnostic workup and support an accurate diagnosis, potentially reducing the need for invasive biopsies or unnecessary surgical interventions.

### 3.6. Reports of Cases

Necrotizing sialometaplasia was first mentioned as a self-limiting inflammatory disorder of salivary gland tissues by Abrams et al. in 1973 [18].

Early reports of parotid gland NS include those by Donath (1979) [6], who documented six cases, and Batsakis and Manning (1987) [1], who described seven cases with associated clinical and pathological findings.

A broader review by Brannon et al. (1991) identified six parotid NS cases within a cohort of 69 total NS cases [5]. A review of the published reports on parotid gland NS revealed a wide age range among patients (17 to 83 years), with an average age of 53.4 years [3].

The current review identified 30 patients diagnosed with necrotizing sialometaplasia of the parotid gland, with a mean age of 49 years (range: 17 to 83 years) and a nearly equal sex distribution. The most common etiology was surgical trauma (20 cases), followed by vascular fragility (4 cases), tumors (1 case), smoking (1 case), and other factors (3 cases).

Clinically, neck swelling was the most frequently reported symptom, often accompanied by pain, pus discharge from the salivary duct, and, in rare cases, pharyngeal swelling, vocal cord paralysis, or neck lymphadenopathy. Lesion sizes ranged from 6 × 10 mm to 40 × 50 mm. Ultrasound, CT, and MRI were commonly used for preoperative evaluation, while fine-needle aspiration biopsy (FNAB) frequently yielded inconclusive results. Histopathological assessment remained the gold standard for diagnosis.

Treatment varied depending on the initial diagnosis. Superficial parotidectomy was performed in cases misdiagnosed as malignancy, and some patients underwent selective neck dissection. Initial histopathological diagnoses included pleomorphic adenoma, mucoepidermoid carcinoma, myoepithelioma, adenoid cystic carcinoma, carcinoma ex pleomorphic adenoma, and lobular sialadenitis. Upon reassessment, NS was confirmed in cases where the lobular architecture remained intact, squamous metaplasia was present, and inflammatory infiltrates were dominant.

We further present the most significant findings in the literature on necrotizing sialometaplasia of the parotid glands (Table 1), expanding and updating the work of Tsuji et al. [3].

### 3.7. Treatment

Necrotizing sialometaplasia typically resolves on its own within 3 to 12 weeks, without the need for specific treatment [5]. The healing process includes regeneration of the damaged salivary tissue, but regeneration of the affected ducts and acini is typically incomplete. During this regenerative phase, ductal and acinar metaplasia, prominent nuclei, frequent mitotic figures, and necrosis may occur, potentially leading to an incorrect diagnosis of malignancy [15].

We recommend further imaging that will demonstrate the spontaneous resolution of the pathology. In nonhealing lesions, diagnostic biopsy or rebiopsy is recommended.

## 4. Clinical Case

A 23-year-old female was referred to our department for a second opinion after a primary surgery for a pT2N0M0 squamous cell carcinoma located in the left parotid gland. The patient was scheduled for radiotherapy in the following week. Six months prior, the patient noticed the lesion as a 0.5 cm lump in the left parotid gland, with progressive growth to approximately 1.5 cm in 2 months. The firm parotid tumor was painful on palpation. The patient had previously been operated on for a deviated septum and chronical adenoiditis (6 months prior), and was diagnosed with Lyme disease one month after the first encounter of the parotid mass. She was a smoker for 6 years (2.1 pack-years).

One month after the initial diagnosis of the parotid lump, she underwent ultrasound investigation, as a result of which the mass was misdiagnosed as an inflammatory lymph node.

**Figure 3:**
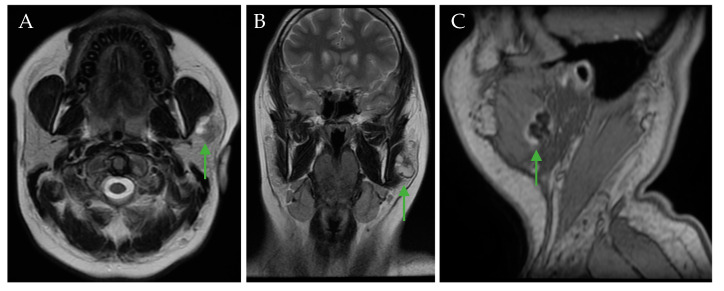
MRI sections showing an apparently well-circumscribed left parotid gland mass (green arrows): axial T2 FSE (fast spin-echo sequence) (**A**), coronal T2 FSE (**B**), and sagittal MPR T1 (Multiplanar reconstruction) (**C**).

Two months later, after the initial oral and maxillofacial exam, CT and MRI imaging revealed a left parotid gland tumor of 20/18/25 mm with a mixed parenchymal and cystic component, with diffusion restriction and inhomogeneous contrast uptake at the level of the solid component (Figure 3).

An open biopsy of the parotid gland tumor confirmed the diagnosis of squamous cell carcinoma of the parotid gland. One month later, the PET-CT showed FDG—avidity within the primary tumor and a few cervical lymph nodes, supporting diagnosis of an ongoing oncologic process.

The patient underwent superficial parotidectomy and selective neck dissection (level II and III). The histopathological report showed a well-differentiated squamous cell carcinoma measuring 20/15 mm (AP/CC), diffusely positive immunohistochemical staining (p63, CK 5/6, CK7), and 30 lymph nodes without metastatic spread.

**Figure 4:**
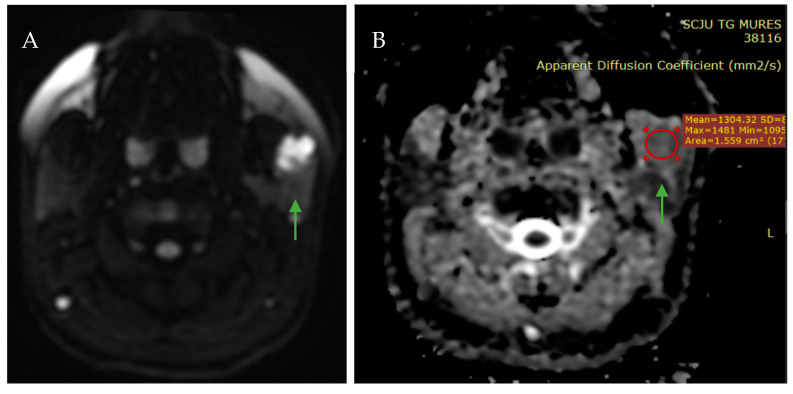
MRI axial sections showing the parotid gland mass (green arrows): (**A**) the axial DWI sequence demonstrating no diffusion restriction, and (**B**) the corresponding ADC map showing a high ADC value (1.3 × 10^−3^ mm^2^/s), the red circle indicates the area where the ADC value was calculated.

Considering the age of the patient, there was a low probability of a primary parotid gland squamous cell carcinoma (3% of all parotid malignancies) [20], as the peak incidence of this is in the seventh and eighth decades of life [19]. Diffusion-Weighted Imaging is an MRI technique that assesses the movement of water molecules within tissues, while the Apparent Diffusion Coefficient quantifies this diffusion, with lower values typically indicating restricted diffusion, as seen in malignancies [21,22,23,24,25]; on DWI sequences, the lesion did not show an diffusion restriction, with the ADC value being approximately 1.3 × 10^−3^ mm^2^/s, raising the possibility of a benign/inflammatory lesion (Figure 4).

Consequently, we decided to repeat the histopathological assessment of the tumor to ensure an accurate diagnosis.

**Figure 5:**
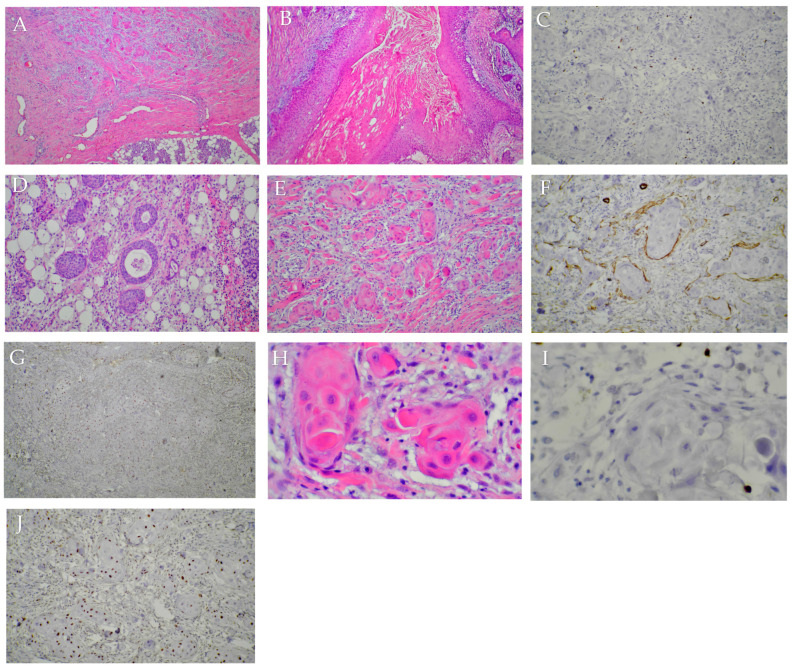
(**A**–**J**) Anatomo-pathological reasoning for a diagnosis of sialometaplasia instead of squamous cell carcinoma: The presence of cystic areas lined by squamous epithelium with focal parakeratosis and focal leukocytic infiltrate, and intraluminal keratin lamellae, is characteristic of necrotizing sialometaplasia. There is immature squamous metaplasia present in numerous ducts located at a distance from the lesion. There is mature epithelium without atypia in the “infiltrative” areas, as well as the extension of squamous nests following a pattern very similar to the tubulo-acinar architecture of the parotid gland. The “buds” of the epithelium protruding from the cyst walls are also a feature of necrotizing sialometaplasia. The stromal infiltrate is chronically inflammatory or fibrous, which is more consistent with necrotizing sialometaplasia than with squamous cell carcinoma. The immunohistochemical staining for p53, calponin, and SMA is consistent with necrotizing sialometaplasia. The Ki-67 proliferation index is increased only at the base of the epithelium in the cyst walls, and is zero in the nests of keratinocytes in the stroma.

Taking into account all the available data, including the patient’s history (Lyme disease and previous ENT interventions), the clinical course of the disease, and imaging characteristics, the second opinion favored a diagnosis of necrotic sialometaplasia (Figure 5).

## 5. Discussion

We considered Lyme disease as a potential contributing factor to local ischemia, which may have influenced the development of necrotizing sialometaplasia. Given that Lyme disease is associated with vasculitis, immune dysregulation, local inflammation, lymphadenopathy, and, in rare cases, parotitis [26], these factors could indirectly increase the risk of NS. Considering that Lyme disease often has a delayed onset of symptomatology following exposure, this timeline discrepancy may reflect the gradual progression of Lyme-associated processes.

The patient’s follow-up included a complete head and neck exam and a US after 1 month and 3 months, followed by an MRI scan at 6 months and 1 year.

At the 1-year follow-up, no recurrence had developed. The patient is currently under strict supervision of the surgical team, and is free of disease.

Anneroth et al. [17] proposed a five-stage classification system for the pathological progression of necrotizing sialometaplasia: infarction (necrotic), sequestration, ulceration, reparative, and healed. Suomalainen et al. [27] observed that the intensity of the inflammatory response can vary across these different stages of NS. The patient’s lesion corresponds to stage 4 (reparative stage) of NS, as evidenced by squamous metaplasia, characterized by the replacement of salivary gland structures with keratinized epithelial nests, intraluminal keratin lamellae, and fibrotic stroma with chronic inflammation.

Necrosis and squamous metaplasia are non-specific findings observed in various inflammatory and neoplastic disorders of the salivary glands. Necrotizing sialometaplasia represents a histologically distinctive entity with specific diagnostic criteria [18].

Pain, swelling, and pus discharge has been reported in several clinical presentations of necrotizing sialometaplasia, and may initially lead to a suspicion of sialolithiasis, especially if the patient seeks medical attention in the early days of parotid swelling. Given that sialolithiasis is a primary indication for sialendoscopy, a careful clinical evaluation, along with sialendoscopic examination, when necessary, may aid in distinguishing between these conditions and preventing misdiagnosis [28].

Differentiating squamous cell carcinoma from necrotizing sialometaplasia can be challenging. In necrotizing sialometaplasia, the lobular architecture of the gland remains intact [29].

Keratocystoma can also be a differential diagnosis for necrotizing sialometaplasia. It is a benign salivary gland tumor characterized by multicystic spaces lined by stratified squamous epithelium, containing keratotic lamellae and focal solid epithelial nests. These cystic and epithelial features may overlap with those seen in sialometaplasia, making keratocystoma an important consideration when diagnosing salivary gland lesions [30].

Given these similarities, the question arises as to whether some cases previously diagnosed as parotid NS might, in fact, represent keratocystomas. This is particularly relevant in cases where RUNX2 rearrangements or IRF2BP2::RUNX2 fusion have been identified, as seen in studies of primary squamous cell carcinoma of the parotid, where certain cases were later reclassified as keratocystoma or squamous cell carcinoma ex-keratocystoma [31].

The case reported by Tsuji et al. (2014) as necrotizing sialometaplasia may have been misdiagnosed, and could instead represent an infarcted Warthin tumor [3]. Slater et al. (2015) raised concerns that key histological features in the case—cystic lumina with eosinophilic necrotic debris, papillary projections within the granulation tissue, and metaplastic stratified squamous epithelium—are more consistent with an infarcted Warthin tumor [32]. These features, along with cystic changes and necrotic oncocytes, are not typically seen in NS. The imaging characteristics of infarcted Warthin tumors and NS also overlap, further complicating diagnosis. Given these challenges, histological verification is essential when diagnosing NS in the parotid gland, in order to distinguish it from cystic or metaplastic lesions such as keratocystoma. Future studies should focus on integrating advanced histopathological and molecular markers to improve diagnostic accuracy [32].

Unlike NS of the minor salivary glands, where ulceration is a common presentation, parotid NS is more frequently associated with post-surgical vascular events or fine-needle aspiration trauma. To date, about 262 cases of NS have been reported. Of these, 8 cases affected the tongue; 37 cases occurred after surgery; and 17 cases showed association with neoplasms. Moreover, the imaging overlap of NS with parotid malignancies underscores the necessity of multiparametric MRI evaluation to guide diagnosis. While NS is predominantly reported in the palate, cases affecting the floor of the mouth, lip, nasal cavity, larynx, and, as recently reported, the tongue, demonstrate that this entity is not restricted to minor salivary glands. Furthermore, recent cases of multifocal NS in the tongue following surgical excision of SCC support the notion that NS is highly associated with local ischemia and surgical trauma, similarly to its occurrence in the parotid gland [33].

Necrotizing sialometaplasia has also been reported in association with various salivary gland tumors, further complicating its diagnosis and differentiation from malignancies. Giunchi and Bulatao [34] described a case where NS developed within a benign mixed tumor (pleomorphic adenoma) of the parotid gland, with histopathological findings showing squamous metaplasia and pseudoepitheliomatous hyperplasia surrounding a necrotic center, mimicking invasive squamous cell carcinoma. Similarly, Gnepp [35] reported NS occurring within a Warthin tumor exhibiting sebaceous differentiation, supporting the theory that ischemia or infarction of salivary gland tumors may trigger NS-like changes. These cases highlight the importance of recognizing NS in the context of coexisting salivary gland tumors to prevent misdiagnosis and unnecessary surgical intervention.

## 6. Conclusions

Necrotizing sialometaplasia, though self-limiting, can be easily misdiagnosed as a malignant tumor. Careful examination of preoperative multiparametric MRI findings is crucial for identifying NS features and preventing unnecessary treatment. If the parotid gland lesion appears more consistent with NS, a conservative “wait and see” approach may be appropriate, given its potential for spontaneous resolution within 3–12 weeks [5]. Accurate diagnosis and effective patient management require a comprehensive approach that integrates clinical, radiologic, and histopathologic findings, emphasizing the critical need to avoid misdiagnosis in complex cases.

## Figures and Tables

**Table 1:** Key findings from parotid gland necrotizing sialometaplasia.

Case	Year	Author	Age (Years)/Sex	Cause	Size	Left/Right/Bilateral Parotid	Clinical Presentation	Preoperative Examination	Treatment	Primary Histologic Diagnosis
1–6	1979	Donath [6]	Mean age of M: 54.5 Mean age of F: 49 Sex ratio: M:F = 1:2	Postoperative vascular injuries (11/13)	0.6–1.0 cm (mean of 6 cases)	NM	Painless ulcer: 70% Nonulcerative mass: 30% Local pain: 26%	NM	Surgical resection	2× pleomorphic adenoma, mucoepidermoid carcinoma, myoepitelioma, adenoid cystic carcinoma, carcinoma ex pleomorphic adenoma, lobular sialadenitis
7–13	1987	Batsakis and Manning [1]								
14–19	1991	Brannon et al. [5]	NM	Postoperative vascular injuries (5/6 cases)	NM	NM	NM	NM	NM	NM
20	2002	Aydin et al. [2]	17/F	Vascular injury	2 cm	Right	Pain Swelling of neck	US/CT FNAB	Superficial parotidectomy	NM
21	2006	Prabhakaran [7]	32/M	Pressure-induced ischaemia	NM	NM	Swelling of neck Pus discharge from parotid duct	Biopsy CT	NM	Parotid abscess
22	2010	Yoshioka [8]	66/M	Malignant lymphoma	NM	NM	Swelling of neck Swelling of pharynx Vocal cord paralysis Neck lymphadenopathy	FNAB	NM	NM
23	2013	Kim et al. [9]	69/F	Vascular injury owing to heavy smoking	3 × 2, 1.5 × 1.5	Bilateral	Swelling of neck	CT FNAB	Superficial parotidectomy	NM
24	2016	Stodulski et al. [10]	35/F	NM	NM	NM	NM	NM	NM	Polymorphous low-grade adenocarcinoma
25	2017	Haen et al. [11]	56/M	Spontaneous hematoma due to overdose of anticoagulant treatment and vascular fragility related to Marfan syndrome	3 × 4 cm	Left	Parotid swelling, inflammation, progressively complete facial nerve paralysis	CT MRI FNAB Open biopsy	NA	Parotiditis Mucoepidermoid carcinoma
26–29	2019	Zhurakivska et al. [12]	45/M	Surgical trauma (FNAB) and/or tumor growth	NM	NM	Parotid gland swelling; slow growth; no facial nerve palsy	US CT FNAB	Total parotidectomy	NM
			51/M		NM	NM	Parotid gland swelling; slow growth; no facial nerve palsy		Superficial parotidectomy	NM
			63/M		NM	NM	Parotid gland swelling		Superficial parotidectomy	NM
			27/M		NM	NM	Parotid gland swelling		Superficial parotidectomy	NM
30	2024	Current report	23/F	Lyme disease	2 × 1.5 cm	Left	Swelling of neck Pain	Biopsy US CT IRM	Superficial parotidectomy and selective neck dissection	Inflammatory lymph node/squamous cell carcinoma

F—female; M—male; NM—not mentioned; US—ultrasonography; FNAB—fine-needle aspiration biopsy; Ga—gallium; NA—not any.

## Abbreviations

The following abbreviations are used in this manuscript:

NS	Necrotizing sialometaplasia
F	Female
M	Male
NM	Not mentioned
US	Ultrasonography
FNAB	Fine-needle aspiration biopsy
GA	Gallium
NA	Not any
DWI	Diffusion-Weighted Imaging
ADC	Apparent Diffusion Coefficient
